# Partially Bio-Based Benzoxazine Monomers Derived from Thymol: Photoluminescent Properties, Polymerization Characteristics, Hydrophobic Coating Investigations, and Anticorrosion Studies

**DOI:** 10.3390/polym16131767

**Published:** 2024-06-21

**Authors:** Arunthip Suesuwan, Natapol Suetrong, Sila Yaemphutchong, Inthikan Tiewlamsam, Kantapat Chansaenpak, Suttipong Wannapaiboon, Nutthawat Chuanopparat, Ladda Srathongsian, Pongsakorn Kanjanaboos, Nalinthip Chanthaset, Worawat Wattanathana

**Affiliations:** 1 Department of Materials Engineering, Faculty of Engineering, Kasetsart University, Ladyao, Chatuchak, Bangkok 10900, Thailand; arunthip.s@ku.th (A.S.); natapol.s@ku.th (N.S.); sila.ya@ku.th (S.Y.); 2 Concord College, Acton Burnell Hall, Acton Burnell, Shrewsbury, Shropshire SY5 7PF, UK; 2335017@concordcollege.org.uk; 3 National Nanotechnology Center, National Science and Technology Development Agency, Thailand Science Park, Pathum Thani 12120, Thailand; kantapat.cha@nanotec.or.th; 4 Synchrotron Light Research Institute, 111 University Avenue, Suranaree, Muang, Nakhon Ratchasima 30000, Thailand; suttipong@slri.or.th; 5 Department of Chemistry, Faculty of Science, Kasetsart University, Ladyao, Chatuchak, Bangkok 10900, Thailand; fscinwc@ku.ac.th; 6 School of Materials Science and Innovation, Faculty of Science, Mahidol University, Nakhon Pathom 73170, Thailand; ladda.sth@gmail.com (L.S.); pongsakorn.kan@mahidol.edu (P.K.); 7 Division of Materials Science, Graduate School of Science and Technology, Nara Institute of Science and Technology, 8916-5 Takayama-cho, Ikoma 630-0192, Nara, Japan; nalin@ms.naist.jp

**Keywords:** benzoxazine, thymol, photoluminescence, polybenzoxazine, hydrophobic coating, anticorrosion

## Abstract

In this work, four thymol-based benzoxazines were synthesized using four primary amines with different chain lengths, namely methylamine, ethylamine, 1-propylamine, and 1-butylamine, which are then named T-m, T-e, T-p, and T-b, respectively. The optical properties of the synthesized thymol-based benzoxazines were examined via the photoluminescent study of their solutions in acetone. The results show that all the prepared benzoxazines emitted blue light with the maximum wavelengths from 425 to 450 nm when irradiated by the excitation wavelengths from 275 to 315 nm. The maximum excitation wavelengths are found to be 275 nm. The polymerization of the thymol-based benzoxazines is triggered by heat treatments with different conditions (160, 180, and 200 °C for 1 h). According to the FTIR results, the heat-curing process introduces a presence of the OH peak, of which intensity increases as the curing temperature increases. Thermal decompositions of thymol-based benzoxazines regarding TGA analyses reveal the enhancement of thermal stability of the benzoxazines with respect to the *N*-substituent chain length, as significantly observed the change in the first thermal decomposition at temperature ranged from 253 to 260 °C. Synthesized benzoxazine derivatives are further employed to coat the substrate, e.g., the glass slides. The investigation of the water contact angle shows that the coating of the benzoxazines onto the surface improves the hydrophobicity of the substrate, resulting in the enlargement of the contact angle from 25.5° to 93.3°. Moreover, the anticorrosion performance of the polybenzoxazine coatings is examined using potentiodynamic polarization techniques. The results illustrate the anticorrosion efficiency of the thymol-based polybenzoxazine up to 99.99%. Both hydrophobic and electrochemical studies suggest the feasibility for employing benzoxazines in anticorrosion coating applications.

## 1. Introduction

Nowadays, the development of high-performance thermoset materials has been significantly expanded as engineering thermosets are applied in a wide range of applications such as space radiation shielding, packaging for electronic devices, and certain everyday-life uses [1]. Polybenzoxazines are classified as one of the phenolic thermosets, possessing distinct physical, thermal, and mechanical properties, so they have gained a great deal of interest from academic and industrial researchers [2,3,4]. Specifically, the polybenzoxazine thermosetting resins possess exceptional characteristics, including a zero percent shrinkage during polymerization [5,6,7,8], low flammability [9,10], a high glass transition temperature [11,12], good dielectric properties [13,14,15,16], and low water absorption [17].

Benzoxazine resins can be easily synthesized by heat-initiated ring opening polymerization without the requirement of any catalysts in case with a sufficient heating temperature. Besides, the benzoxazine monomers are simply prepared through the one-pot Mannich reactions of three reagents, namely phenolic derivatives, primary amines, and formaldehyde [18]. The high variety and availability of the starting materials, especially phenols and primary amines, provide the advantages for property-tunable molecular designs. Therefore, benzoxazines and polybenzoxazines with desired properties can be tailored. Even though the feedstocks for synthesizing benzoxazines are ubiquitous, most are derived from petroleum-based chemicals that are currently being used up [19,20]. Therefore, the endeavor to search for potential bio-derived resources as renewable raw materials is necessary for long-term sustainability.

Among the three reactants for benzoxazine syntheses, both the phenol and amine derivatives are commonly changed to bio-derived compounds. The prevailing bio-based primary amines are furfurylamine [21,22,23], stearylamine [23], dehydroabietylamine [24], etc. In the case of bio-derived phenols, the compounds obtained from plants are of interest. For instance, cardanol [25], urushiol [26], guiacol [23], resorcinol [27], chavicol [28], eugenol [29], coumarin [30], rosin [24], vanillin [31,32], lignin [33], and chalcone [34] are reported as substitutes for petroleum-based phenols. The usage of bio-derived feedstock is not only known for solving environmental issues but also for the improvement of various physicochemical properties and thermal stability [35,36].

Among the plant-based phenols, thymol is one of the most promising candidates for industrial-scale production due to its relatively low toxicity and cost. Even though several properties of benzoxazines and polybenzoxazines have been reported up to now, their photoluminescent properties are rarely examined. Our group has reported some of the luminescence characteristics of some benzoxazines derived from petroleum-based phenol [37,38,39]. The objective of this present work is to prepare partially bio-based benzoxazine monomers from thymol and primary amines with different alkyl chains. The chemical structures of the synthesized benzoxazines are confirmed via FTIR, ^1^H NMR, and ^13^C NMR spectroscopies. The thermal characteristics and ring-opening polymerization behaviors of bio-benzoxazine are examined using differential scanning calorimetry (DSC), while the thermal stability of the benzoxazines is investigated through thermogravimetric analysis (TGA). Moreover, the effect of *N*-substituent groups on the photoluminescent properties of benzoxazines is studied. Apart from the optical properties, the hydrophobicity anticorrosion properties of the synthesized thymol-based benzoxazines are investigated.

## 2. Experimental

### 2.1. Materials

The required chemicals and solvents for the syntheses are as follows: thymol (99% purity, ChemSupply Australia, Gillman, SA, Australia), paraformaldehyde (95% purity, Sigma Aldrich, St. Louis, MO, USA), methylamine (40% aqueous solution, Merck, Rahway, NJ, USA), ethylamine (70% aqueous solution, Merck), propylamine (99% purity, Merck), butylamine (99% purity, Merck), sodium hydroxide (97%, Kemaus, Cherrybrook, NSW, Australia), anhydrous sodium sulfate (99%, Daejung, Sasang-gu, Busan, South Korea), ethanol (99.9%, RCI Labscan, Bangkok, Thailand), and dichloromethane (99.8%, RCI Labscan, Bangkok, Thailand). All the chemicals were used as received without additional purifications.

### 2.2. Syntheses of Thymol-Based Benzoxazine Monomers

**Scheme 1:**
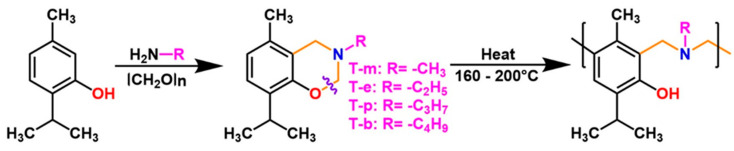
The reaction scheme for the benzoxazine synthesis as well as the ring-opening polymerization reaction.

For the synthesis of benzoxazine monomers, thymol was used as a phenol starting material, and the amines were varied by using methylamine, ethylamine, propylamine, and butylamine. Scheme 1 shows the reactions involved in the synthesis of the benzoxazine monomers, which are similar to the methods described in the literature [37,38,39]. In the case of methylamine-based benzoxazine, thymol (22.53 g, 150 mmol), paraformaldehyde (9.46 g, 315 mmol, excess), and methylamine (12.9 mL, 40% in aqueous solution, 150 mol) were dissolved in 80 mL of ethanol and stirred. Then, the mixture was heated until boiling and maintained for 3 h. After the reaction finished, the mixture turned into a clear-yellow solution. Next, 50 mL of dichloromethane was added to the solution of the crude product, and then the solution was extracted with 3 N of NaOH and deionized water to remove unreacted reactants. The organic layer was separated from the aqueous layer using a separatory funnel and then dried with anhydrous Na_2_SO_4_. The organic solvents were evaporated by a rotary evaporator. The final product of thymol-methylamine-based benzoxazine was labeled as T-m. The other three benzoxazine monomers from ethylamine, propylamine, and butylamine were obtained by the same procedure with the same mole ratio of thymol: paraformaldehyde: amine of 1:2.1(slight excess):1. The nomenclature of the benzoxazine monomers from ethylamine, propylamine, and butylamine were T-e, T-p, and T-b, respectively.

### 2.3. The Characterization of the Thymol-Based Benzoxazines

To confirm the structure of the synthesized benzoxazines, FTIR, ^1^H NMR, and ^13^C NMR were carried out. FTIR spectra were recorded using a Bruker Alpha FTIR spectrometer (Billerica, MA, USA). One hundred scans of the samples and backgrounds were applied to all the measurements. The spectral resolution was ±2 cm^−1^. A nuclear magnetic resonance spectrometer (Bruker AVANCE III 400 MHz) was used to measure both the ^1^H and the ^13^C NMR spectra of the synthesized thymol-based benzoxazines. The samples were dissolved in CDCl_3_ prior to the NMR experiments.

The thermal properties of the thymol-based benzoxazines were investigated using thermogravimetric analysis (TGA, PerkinElmer TGA 4000 model, Waltham, MA, USA) and differential scanning calorimetry (DSC, NETZSCH 214 Polyma model, Selb, Bayern, Germany). During the measurements, nitrogen was purged at a flow rate of 60 mL/min for both the TGA and DSC. The heating rate was set to 20 °C/min.

T-m: ^1^H NMR (400 MHz, CDCl_3_): δ 7.04 (d, *J* = 7.7 Hz, 1H), 6.77 (d, *J* = 7.8 Hz, 1H), 4.80 (d, *J* = 0.8 Hz, 2H), 3.90 (s, 2H), 3.31 (hept, *J* = 7.0 Hz, 1H), 2.66 (s, 3H), 2.18 (s, 3H), 1.26 (dd, *J* = 7.0, 1.4 Hz, 6H); ^13^C NMR (100 MHz, CDCl_3_): δ 150.8, 133.7, 133.3, 123.6, 121.6, 118.0, 82.9, 51.1, 40.2, 26.2, 22.7, 18.0. (Yield 69%).

T-e: ^1^H NMR (400 MHz, CDCl_3_): δ 7.03 (d, *J* = 7.8 Hz, 1H), 6.76 (d, *J* = 7.8 Hz, 1H), 4.89 (s, 2H), 3.95 (s, 2H), 3.28 (hept, *J* = 6.9 Hz, 1H), 2.86 (q, *J* = 7.1 Hz, 2H), 2.19 (s, 3H), 1.25 (t, *J* = 7.2 Hz, 3H), 1.25 (d, *J* = 6.9 Hz, 6H); ^13^C NMR (100 MHz, CDCl_3_): δ 151.4, 133.6, 133.2, 123.5, 121.5, 118.4, 81.1, 48.7, 45.9, 26.2, 22.7, 18.0, 13.3. (Yield 70%).

T-p: ^1^H NMR (400 MHz, CDCl_3_): δ 7.03 (d, *J* = 7.8 Hz, 1H), 6.75 (d, *J* = 7.8 Hz, 1H), 4.87 (s, 2H), 3.94 (s, 2H), 3.28 (hept, *J* = 13.9, 6.9 Hz, 1H), 2.81–2.66 (m, 2H), 2.18 (s, 3H), 1.75–1.57 (m, 2H), 1.25 (d, *J* = 6.9 Hz, 6H), 1.00 (t, *J* = 7.4 Hz, 3H); ^13^C NMR (100 MHz, CDCl_3_): δ 151.4, 133.6, 133.2, 123.4, 121.4, 118.5, 81.7, 53.9, 49.0, 26.1, 22.7, 21.3, 18.0, 11.7. (Yield 78%).

T-b: ^1^H NMR (400 MHz, CDCl_3_): δ 7.04 (d, *J* = 7.8 Hz, 1H), 6.76 (d, *J* = 7.7 Hz, 1H), 4.88 (s, 2H), 3.94 (s, 2H), 3.28 (hept, *J* = 7.0 Hz, 1H), 2.85–2.71 (m, 2H), 2.19 (s, 3H), 1.70–1.57 (m, 2H), 1.43 (dq, *J* = 14.6, 7.3 Hz, 2H), 1.26 (d, *J* = 6.9 Hz, 6H), 1.00 (t, *J* = 7.3 Hz, 3H); ^13^C NMR (100 MHz, CDCl_3_) δ 151.4, 133.6, 133.2, 123.4, 121.4, 118.5, 81.7, 51.6, 49.1, 30.3, 26.2, 22.7, 20.5, 18.0, 14.0. (Yield 72%).

Note that the ^1^H and ^13^C NMR spectra of all the synthesized benzoxazines are illustrated in the Supplementary Materials (Figures S1–S8).

### 2.4. Photoluminescence Studies

The photoluminescence behaviors of the benzoxazine monomers (T-m, T-e, T-p, and T-b) were investigated using a fluorescence and absorbance spectrometer (Duetta, Horiba Scientific, Kyoto, Japan). The samples were dissolved in acetone to make a solution prior to measurement. The excitation wavelength range was set to 275–315 nm, while the emission wavelength range was set to 300–700 nm. Both of the slits for excitation and emission were set to 10 nm, while the integration time was 6 s.

### 2.5. The Deposition of Polybenzoxazine Films

The thymol-based polybenzoxazines, [poly(T-m), poly(T-e), poly(T-p), and poly(T-b)], were prepared through ring-opening polymerization triggered by thermal treatment at different conditions (Scheme 1). Prior to the polymerization process, glass slides were cleaned with acetone and dried in an oven. The solutions of thymol-based benzoxazine monomers were prepared by dissolving 0.2 g of each benzoxazine monomer in 1 mL of acetone. A 300 μL of each prepared solution was dropped and spin-coated onto the glass slides using a spin speed of 1500 rpm for 15 s. The coated glass slides were subsequently heated in the oven at 160, 180, and 200 °C for 1 h to undergo ring-opening polymerization.

### 2.6. Water Contact Angle Measurement

In this study, the contact angles of the coatings were measured to determine the hydrophobicity of the thermoset films cured at different temperatures. The volume of the liquid used for each measurement was 0.5 μL, and three different dropping positions were taken for the contact angle test of each sample. The sessile drop and tangent searching fitting modes were applied to fit the contact angle using FAMAS software.

### 2.7. Anticorrosion Study

The SS304 size 20 × 20 × 1.2 mm was cleaned with acetone to remove grease and foreign matter before being coated with the prepared thymol-based benzoxazines. The coating of thymol-based benzoxazines on the SS304 surfaces was performed by dissolving the thymol-based benzoxazines in an acetone solvent at a concentration of 0.2 g/mL. The prepared solution was dropped onto the SS304 surfaces in amounts of approximately 300 µL through a spin-coating process at 1500 rpm for 30 s and then cured at 160, 180, and 200 °C for 1 h.

Electrochemical investigations were analyzed by the electrochemical software PSTrace version 5.7. The standard three-electrode system (i.e., an SS304 plate with and without the polybenzoxazine coatings was used as the working electrode, Ag/AgCl in 3.0 M of a KCl electrode was used as the reference electrode, and platinum wire was used as the counter electrode) was immersed in 1.0 M of an NaCl electrolyte solution throughout the measurement. The scanning potential of the potentiodynamic polarization (LSV) was scanned at a scan rate of 1 mV·s^−1^.

## 3. Results and Discussion

### 3.1. The Thermal Properties of the Thymol-Based Benzoxazines

**Figure 1:**
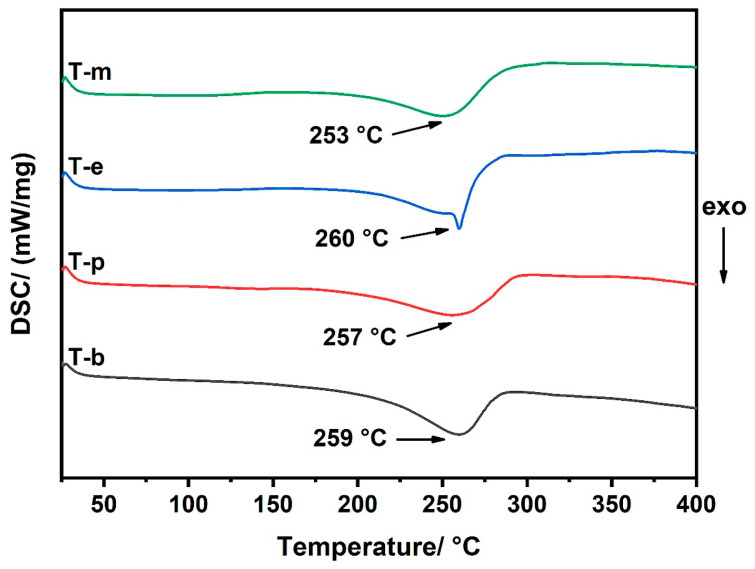
The DSC curves of the synthesized thymol-derived benzoxazine monomers indicating their corresponding curing temperatures.

**Figure 2:**
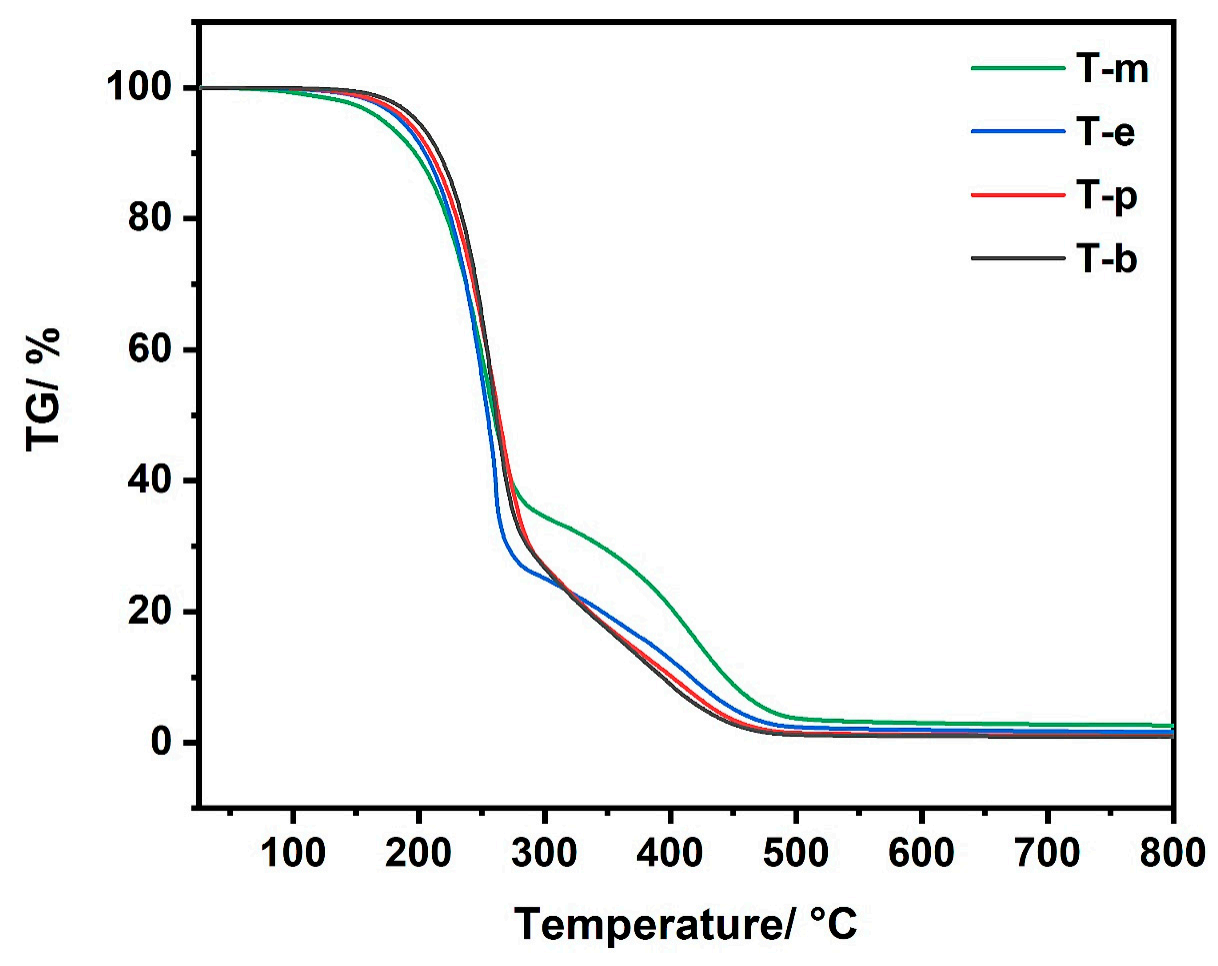
The TGA curves of the synthesized thymol-derived benzoxazine monomers.

The thermal properties of the thymol-based benzoxazines are investigated by DSC (Figure 1) and TGA (Figure 2). According to the DSC curves (Figure 1), no endothermic peak is evidently observed for all the monomers, implying that the benzoxazine monomers are amorphous [40]. However, the exothermic peak corresponding to the ring-opening polymerization (Scheme 1) is noticed [40,41,42,43,44]. The curing temperatures of the benzoxazines are in the range from 253 °C to 260 °C, which are in line with the curing temperatures of the benzoxazine monomers with *N*-aliphatic substituents reported in the literature [40,44]. The curing temperature of T-m is lower than that of the other benzoxazines due to its shortest *N*-alkyl chain among others, while the curing temperatures of T-e, T-p, and T-b are pretty similar (Table 1).

In addition to the curing behaviors, the thermal stabilities of the benzoxazines are investigated using TGA. It is observed that all the synthesized benzoxazines exhibit two-stage weight loss as seen from Figure 2 and Table 2. There is no weight loss below 100 °C, implying that the organic solvent and water have been completely removed [45]. In the first stage of decomposition, the temperature of T-m (253 °C) is slightly lower than that of the other benzoxazines (259–260 °C), but the weight loss of T-m in the first decomposition stage is around 10% lower than that of the other benzoxazines. The first weight loss is considered to be the most significant step since its percentage is in the range of 71% to 83%. The second weight loss occurs at the temperature in the range of 350 °C to 422 °C, with the mass percentage around 16% to 26%. After two weight losses, the extent of weight decomposition is 97% to 99%, where the decomposition extent is in the order of T-m < T-e < T-p < T-b. The third decomposition at the temperature range of 600 to 627 °C is negligible compared to the first two decomposition steps, supporting the two-step thermal degradation of the synthesized thymol-based benzoxazine monomers. Moreover, the amount of coke residue after 800 °C has the same tendency as the decomposition extent.

### 3.2. Photoluminescent Properties of the Thymol-Based Benzoxazines

**Figure 3:**
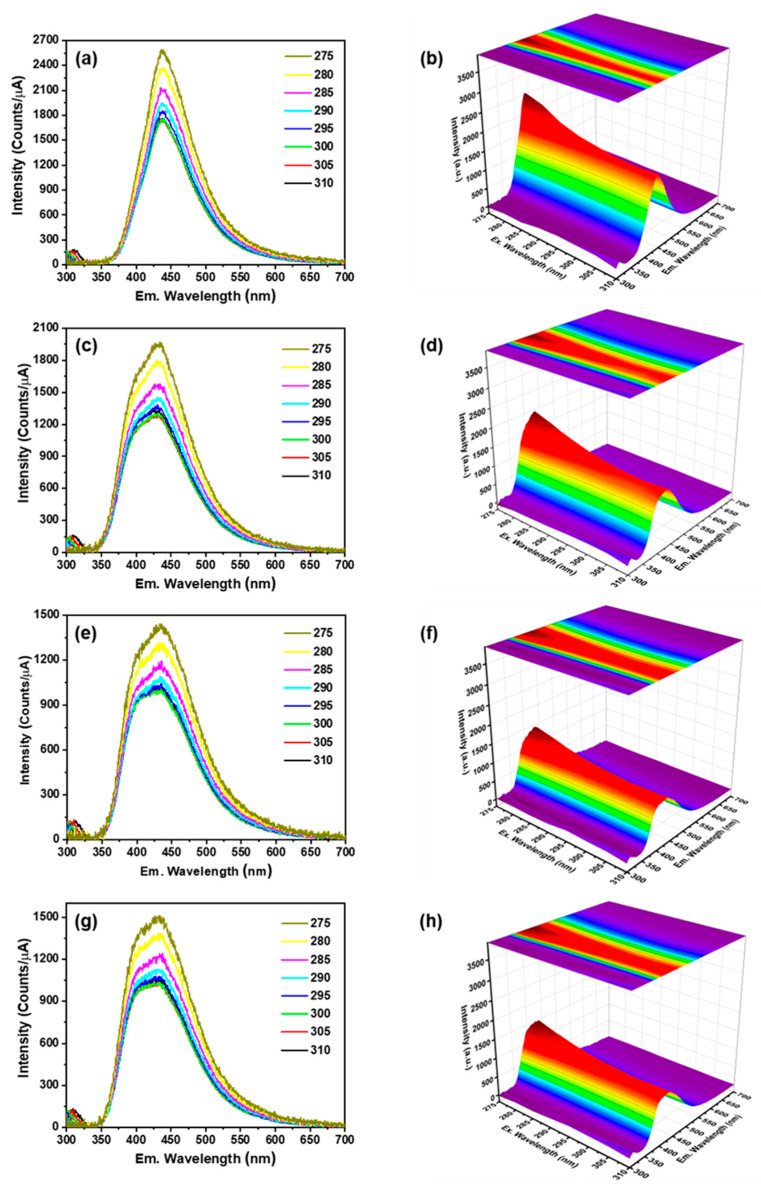
The emission spectra as well as the corresponding excitation–emission matrices using the excitation wavelength range of 275–315 nm of (**a**,**b**) T-m, (**c**,**d**) T-e, (**e**,**f**) T-p, and (**g**,**h**) T-b.

**Figure 4:**
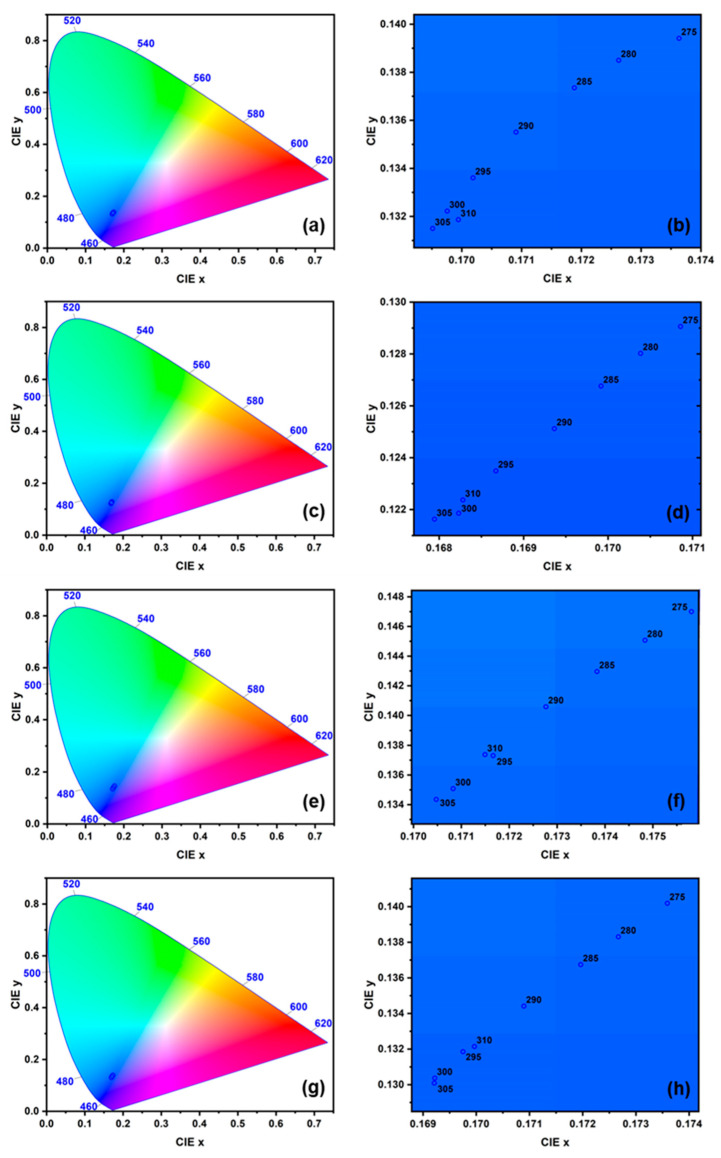
The CIE plot (left) together with the close look at the color coordinates (right) for (**a**,**b**) T-m, (**c**,**d**) T-e, (**e**,**f**) T-p, and (**g**,**h**) T-b.

The photoluminescent properties in terms of emission spectra and excitation–emission matrices for the thymol-based benzoxazines are investigated by exciting their solutions in acetone within the excitation wavelength range from 275 to 310 nm (Figure 3). It is found that all the benzoxazines emit luminescent light in the range of 425 to 450 nm, which corresponds with light blue color. For all the benzoxazine studied, the emission intensity gradually lowers when the excitation wavelength changes from 275 to 300 nm, but the intensity is stable after a further increase in the excitation wavelength from 300 to 310 nm. When closely considered, the solution of T-m gives the highest intensity compared to others with no shoulder peak, but the shoulder peak centered around 400 nm is observed in the emission spectra of the other derivatives (T-e, T-p, and T-b). The intensity of the major emission peak (~450 nm) is weakened when the chain length of the substituent group increases. This is because the longer chain length has higher mobility, which can lead to the non-radiative relaxation of the excited electrons. However, the luminescent intensity of T-p and T-b are pretty similar. The color coordinate [46,47,48] of the thymol-based benzoxazines is calculated from the emission spectra at different excitation wavelengths, as shown in Figure 4. It is recognized that all the benzoxazines give a blue luminescence when excited by the photon with wavelengths from 275 to 300 nm. The shifts in the CIE (x, y) coordinates are due to the different intensities of the shoulder and major peaks.

### 3.3. Monitoring the Ring-Opening Polymerization of the Thymol-Based Benzoxazines Using FTIR Spectroscopy

**Figure 5:**
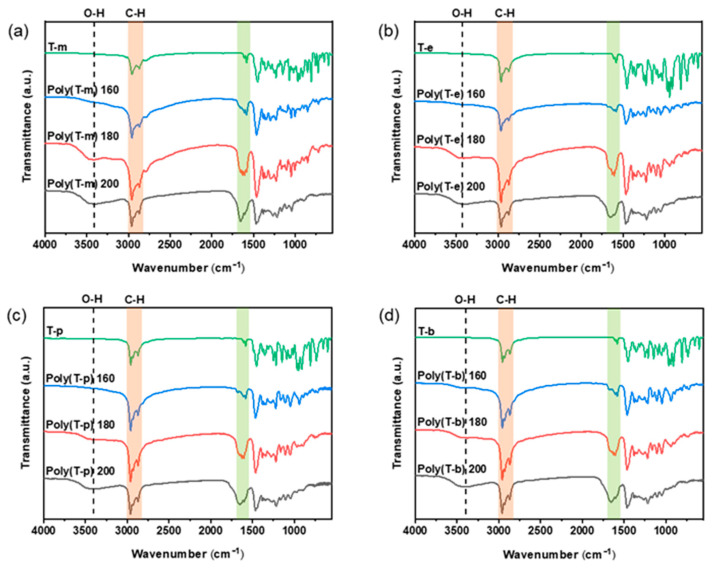
The FTIR spectra of the thymol-based benzoxazines (**a**) T-m, (**b**) T-e, (**c**) T-p, and (**d**) T-b, together with the FTIR spectra of their corresponding thermally-treated products under different conditions (160, 180, or 200 °C for 1 h).

**Figure 6:**
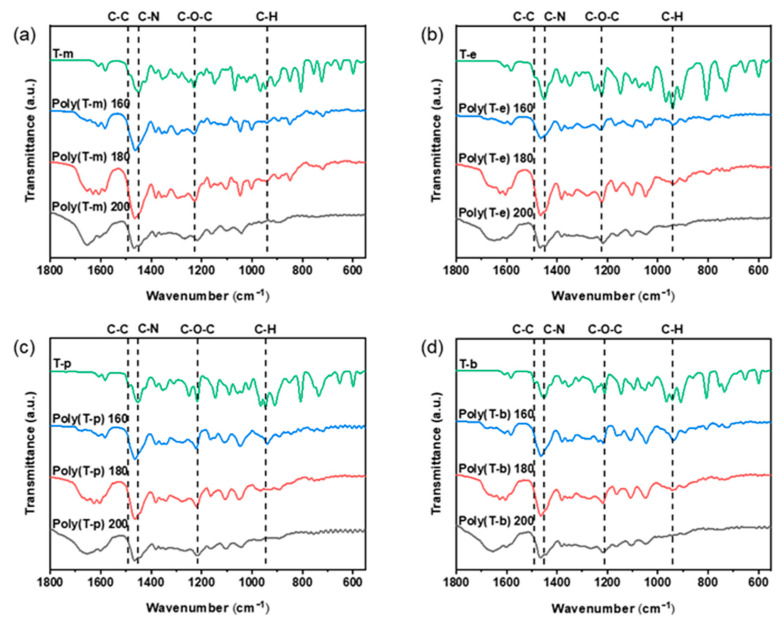
The fingerprint regions of the FTIR spectra of the thymol-based benzoxazines (**a**) T-m, (**b**) T-e, (**c**) T-p, and (**d**) T-b, together with the FTIR spectra of their corresponding thermally treated products under different conditions (160, 180, or 200 °C for 1 h).

The FTIR spectra of all the thymol-based benzoxazines, as well as their corresponding thermally-cured products, are illustrated in Figure 5 and Figure 6. For the spectra of the benzoxazine monomers, four characteristic peaks of the oxazine ring at the wavenumbers around 805, 940, 1220, and 1490 cm^−1^ are observed, which are attributed to the C–N–C vibration, the C_benzene_-H_benzene_ out-of-plane deformation, the C–O–C asymmetric stretching, and the tangential C–C stretching vibration [49], respectively. The peak found at around 1350 cm^−1^ is due to the tetra-substituted benzene moiety [44]. Furthermore, the absence of a thymol O–H peak at 3200 cm^−1^ confirms the complete formation of benzoxazines [45]. Apart from the peaks regarding the benzoxazine core structure, the peaks involved in the side chains are also found. For example, in the case of T-m, there are two peaks at 2957 and 2869 cm^−1^ due to asymmetric and symmetric CH_3_ stretching vibrations, respectively [46]. For other derivatives, two additional peaks responsible for the asymmetric and symmetric CH_2_ stretching vibrations are noticed [50].

When the benzoxazine monomers are subjected to thermal treatment at different conditions (160, 180, 200 °C for 1 h), the significant features of the FTIR spectra are changed as the oxazine ring usually undergoes ring-opening reaction at temperatures in the range from 160–220 °C [51]. For instance, the intensities of the characteristic peaks of the oxazine ring are disappearing. Moreover, the broad peak centered at 3380 cm^−1^ can be assigned to the presence of the OH functional group formed by ring-opening polymerization. The OH peak of the cured products of the benzoxazines is found at a significantly higher wavenumber than the OH of thymol as the OH groups of the polybenzoxazines are capable of forming intramolecular O–H···N and intermolecular O–H···O hydrogen bonds [7,50,52,53]. It is further recognized that the intensity of the OH band increases as the curing temperature increases, as seen in Figures S9–S12 and Tables S1–S4. This implies that the extent of the ring-opening polymerization reaction is dependent on temperature, which corresponds to the literature [54]. Apart from the formation of the OH peak, the peak at 1600 cm^−1^ is shifted to 1650 cm^−1^ as elevating the temperature. This implies the formation of a keto tautomer containing the characteristic C=O bond from the presence of OH group attached to the benzene ring.

### 3.4. Wettability Study

**Figure 7:**
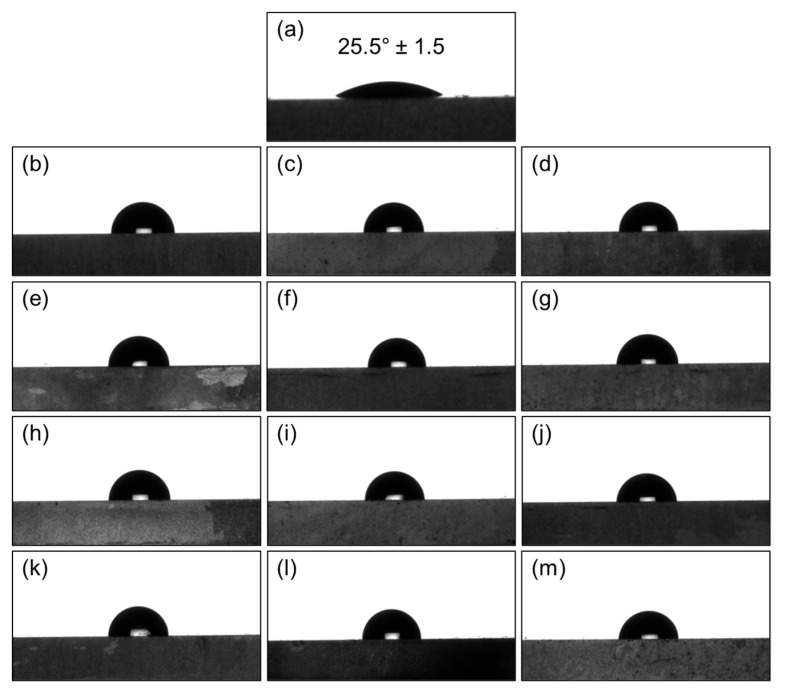
The appearance of a water droplet on the following substrates: (**a**) the uncoated glass slide and glass slides coated with (**b**) T-m cured at 160 °C, (**c**) T-m cured at 180 °C, (**d**) T-m cured at 200 °C, (**e**) T-e cured at 160 °C, (**f**) T-e cured at 180 °C, (**g**) T-e cured at 200 °C, (**h**) T-p cured at 160 °C, (**i**) T-p cured at 180 °C, (**j**) T-p cured at 200 °C, (**k**) T-b cured at 160 °C, (**l**) T-b cured at 180 °C, and (**m**) T-b cured at 200 °C.

Figure 7 illustrates the images of water contact angles of the glass slide surface with and without coating with poly(T-m), poly(T-e), poly(T-p), and poly(T-b) at different curing temperatures. The values of the contact angles with respect to Figure 7 are listed in Table 3. The uncoated glass slide has a contact angle of 25.5° ± 1.5°, while the contact angles of the coated substrates are observed in the range of 89.0° ± 0.4° to 93.6° ± 0.5°. Although polybenzoxazines contain several OH groups, the benzoxazine coatings can decrease the water affinity of the substrates due to the existence of intramolecular hydrogen bonds in polybenzoxazines [7,50,52,53]. The formation of the intramolecular O–H···N hydrogen bonding within the polybenzoxazine molecules could lower the surface free energy, leading to hydrophobicity enhancement [45]. Therefore, the contact angle of the polybenzoxazine-coated glass slide is notably higher than that of the uncoated glass slide. Moreover, the hydrophobicity of the polybenzoxazine coatings can be improved by adjusting the *N*-alkyl group. Herein, the hydrophobicity is slightly increased with respect to the chain length, as seen in Table 3. Additionally, the contact angle tends to increase with the curing temperature, which is in line with the literature [54].

### 3.5. Anticorrosion Study

**Figure 8:**
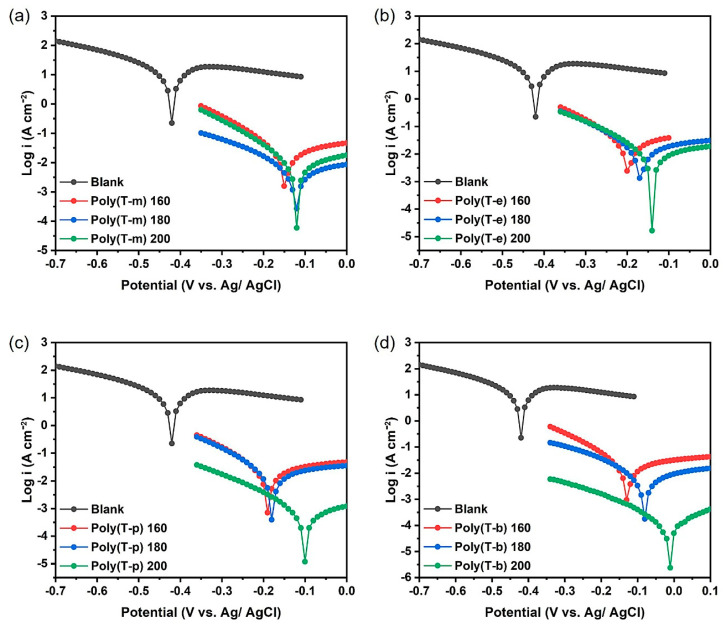
The Tafel polarization curves of SS304 without (blank) and with polybenzoxazine coatings: (**a**) T-m cured at 160 °C, 180 °C, and 200 °C; (**b**) T-e cured at 160 °C, 180 °C, and 200 °C; (**c**) T-p cured at 160 °C, 180 °C, and 200 °C; and (**d**) T-b cured at 160 °C, 180 °C, and 200 °C, immersing in 1 M NaCl solution.

The anticorrosion behavior of thymol-based benzoxazines is studied using potentiodynamic polarization. Figure 8 shows the Tafel plots of the bare SS304 plate and the SS304 coated with thymol-based benzoxazines at different curing temperatures (160 °C, 180 °C, and 200 °C) in 1.0 M of a NaCl solution, which can then be used to calculate the corrosion potential (E_corr_) and corrosion current density (I_corr_) from the anodic (β_a_) and cathodic (β_c_) slope analysis. The obtained parameters are listed in Table 4. The corrosion rate (CR) is calculated from Equation (1) [55].

(1) $$\mathrm{CR}=\frac{I_{\mathrm{corr}}\times K\times \mathrm{EW}}{\rho A}$$

The variable descriptions are as follows: the corrosion rate constant (K) = 3272 mm year^−1^ [56], the equivalent weight (EW) = 25.12 gmol^−1^ [56], the material density (ρ) = 7.94 g cm^−3^, and the surface area (A) = 1.0 cm^2^ for SS304. In addition, the anticorrosion efficiency (%IE) of the thymol-based benzoxazine coatings can be calculated from Equation (2).

(2) $$\mathrm{IE}(\%)=\frac{I_{\mathrm{corr}}{-I}_{\mathrm{corr}}^{0}}{I_{\mathrm{corr}}}\times 100$$

I_corr_ and $I_{\mathrm{corr}}^{0}$ are the corrosion current values in the absence and the presence of the coatings, respectively.

In general, a faster corrosion rate corresponds to a more negative E_corr_ value, resulting in more I_corr_, whereas a more positive E_corr_ value and less I_corr_ means a slower corrosion process [57,58]. As shown in Figure 8 and Table 4, both the corrosion currents decreased by up to four orders of magnitude when the SS304 surface was coated with thymol-based benzoxazine (poly(T-b)) cured at 200 °C for 1 h). Its IE% was found to be 99.99%. For all the thymol-based benzoxazines, the anticorrosion efficiencies increase with the curing temperature in a similar trend. This is because the higher curing temperature enhances a complete polybenzoxazine film formation, as seen from the FTIR results. The anticorrosion performance of the polybenzoxazine films is commenced by the adhesion of passive benzoxazine films onto the SS304 surface, the reaction inhibiting at anodic and cathodic sites, and the decreased corrosion current density, consecutively [42,43].

## 4. Conclusions

Four thymol-based benzoxazines were synthesized by a one-pot Mannich reaction of thymol, paraformaldehyde, and primary amines (methylamine, ethylamine, 1-propylamine, and 1-butylamine). All the synthesized thymol-based benzoxazines exhibit blue emissions, with maximum wavelengths around 425–450 nm. Within the excitation wavelength range of 275–315 nm, the maximum excitation wavelengths are found to be 275 nm. TGA analyses reveal that thermal stability increases with the prolongation of the *N*-substituent chain length. The ring-opening polymerization of the thymol-based benzoxazines is observed when heated at 160, 180, and 200 °C for 1 h. FTIR spectroscopy is used to monitor the ring-opening polymerization by the disappearance of some benzoxazine core vibrations and the formation of the OH peak at 3380 cm^−1^. The coating process of the thymol-based benzoxazines was successfully done using a spin-coating method. The hydrophobicity of the surface is improved by the polybenzoxazine coatings due to the existence of intramolecular hydrogen bonds, which decrease the surface’s free energy. Hydrophobicity can be tailored by changing the alkyl side chain and curing temperature. The potentiodynamic polarization study reveals that the polybenzoxazine coatings improve the anticorrosion in terms of the IE% up to 99.9% (in the case of the poly(T-b) coating cured at 200 °C for 1 h). Additionally, the anticorrosion performance relies more on the curing temperature than the prolongation of *N*-substituent size chain. The results show the possibility of employing thymol-derived benzoxazine as an anticorrosion coating.

## Figures and Tables

**Table 1:** The curing temperatures and corresponding enthalpy changes (ΔH) of the thymol-based benzoxazine monomers.

Benzoxazine Monomer	T_cure_ (°C)	ΔH (mJ)
T-m	253	−4821
T-e	260	−4029
T-p	257	−2490
T-b	259	−2380

**Table 2:** The decomposition temperatures and corresponding weight losses of the thymol-based benzoxazine monomers.

Benzoxazine Monomer	First Stage		Second Stage		Third Stage		Residue at 800 °C (%)
	T (°C)	Weight Loss (%)	T (°C)	Weight Loss (%)	T (°C)	Weight Loss (%)	
T-m	253	70.62	422	26.36	602	0.34	2.67
T-e	261	80.52	415	17.52	615	0.35	1.61
T-p	261	82.30	350	16.46	600	0.14	1.09
T-b	260	82.61	399	16.38	627	0.10	0.91

**Table 3:** The contact angle of the glass slides coated with different thymol-based benzoxazines cured at temperatures in the range of 160–200 °C for 1 h. Note that the contact angle of the uncoated glass slide is 25.5° ± 1.5°.

Benzoxazine Monomer	Contact Angle (°) for Polybenzoxazine Cured at the Temperature of		
	160 °C	180 °C	200 °C
T-m	89.8 ± 0.2	91.3 ± 0.5	91.1 ± 0.2
T-e	89.0 ± 0.4	92.0 ± 0.4	91.7 ± 0.4
T-p	90.3 ± 0.4	92.6 ± 0.3	92.3 ± 0.3
T-b	91.7 ± 0.4	92.9 ± 0.4	93.6 ± 0.5

**Table 4:** The potentiodynamic polarization parameters of SS304 without (blank) and with polybenzoxazine coatings in 1 M NaCl solution.

Sample	Tafel Slopes (mV Dec^−1^)		−E_corr_ (mV)	I_corr_ (µA cm^−2^)	Corrosion Rate (mmy^−1^) × 10^5^	IE (%)
	β_a_	β_c_				
Blank	209	194	399	13,160	8000	-
Poly(T-m) 160	273	108	143	162.4	16.81	98.77
Poly(T-m) 180	698	160	125	58.27	6.03	99.56
Poly(T-m) 200	94	82	111	36.55	3.78	99.72
Poly(T-e) 160	107	149	164	207.8	21.51	98.42
Poly(T-e) 180	341	127	163	125.1	12.95	99.05
Poly(T-e) 200	22	64	118	16.21	1.68	99.88
Poly(T-p) 160	450	130	183	210.8	21.82	98.40
Poly(T-p) 180	462	133	172	167.1	17.29	98.73
Poly(T-p) 200	156	110	90	3.97	0.41	99.97
Poly(T-b) 160	503	135	123	180.5	18.69	98.63
Poly(T-b) 180	220	140	71	44.47	4.6	99.66
Poly(T-b) 200	172	163	3	1.07	0.11	99.99

## Data Availability

Data are contained within the article and Supplementary Materials.

## Supplementary Materials

The following supporting information can be downloaded at: https://www.mdpi.com/article/10.3390/polym16131767/s1, Figure S1: ^1^H NMR spectrum of T-m; Figure S2: ^13^C NMR spectrum of T-m; Figure S3: ^1^H NMR spectrum of T-e; Figure S4: ^13^C NMR spectrum of T-e; Figure S5: ^1^H NMR spectrum of T-p; Figure S6: ^13^C NMR spectrum of T-p; Figure S7: ^1^H NMR spectrum of T-b; Figure S8: ^13^C NMR spectrum of T-b; Figure S9: The O–H peak area of (a) T-m, (b) T-m cured at 160 °C, (c) T-m cured at 180 °C, and (d) T-m cured at 200 °C; Figure S10: The O–H peak area of (a) T-e, (b) T-e cured at 160 °C, (c) T-e cured at 180 °C, and (d) T-e cured at 200 °C; Figure S11: The O–H peak area of (a) T-p, (b) T-p cured at 160 °C, (c) T-p cured at 180 °C, and (d) T-p cured at 200 °C; Figure S12: The O–H peak area of (a) T-b, (b) T-b cured at 160 °C, (c) T-b cured at 180 °C, and (d) T-b cured at 200 °C; Table S1: The O–H peak area of (a) T-m, (b) T-m cured at 160 °C, (c) T-m cured at 180 °C, and (d) T-m cured at 200 °C; Table S2: The O–H peak area of (a) T-e, (b) T-e cured at 160 °C, (c) T-e cured at 180 °C, and (d) T-e cured at 200 °C; Table S3: The O–H peak area of (a) T-p, (b) T-p cured at 160 °C, (c) T-p cured at 180 °C, and (d) T-p cured at 200 °C; Table S4: The O–H peak area of (a) T-b, (b) T-b cured at 160 °C, (c) T-b cured at 180 °C, and (d) T-b cured at 200 °C.
