# Supplementary material for: Partially Bio-Based Benzoxazine Monomers Derived from Thymol: Photoluminescent Properties, Polymerization Characteristics, Hydrophobic Coating Investigations, and Anticorrosion Studies
Source: Polymers (Basel). 2024 Jun 21;16(13):1767. doi: 10.3390/polym16131767 (PMC11244396; doi:10.3390/polym16131767)
Supplement: Supplementary file 1 [file polymers-16-01767-s001.zip › polymers-2910904-supplementary.pdf]

polymers

MDPI

Supplementary Materials

Partially Bio-Based Benzoxazine Monomers Derived from Thymol: Photoluminescent Properties, Polymerization Characteristics, Hydrophobic Coating Investigations, and Anticorrosion Studies

Arunthip Suesuwan [1], Natapol Suetrong [1], Sila Yaemphutchong [1], Inthikan Tiewlamsam [2], Kantapat Chansaenpak [3], Suttipong Wannapaiboon [4], Nutthawat Chuanopparat [5], Ladda Srathongsian [6], Pongsakorn Kanjanaboos [6], Nalinthip Chanthaset [7] and Worawat Wattanathana [1,*]

[1] Department of Materials Engineering, Faculty of Engineering, Kasetsart University, Ladyao, Chatuchak, Bangkok 10900, Thailand; arunthip.s@ku.th (A.S.); natapol.s@ku.th (N.S.); sila.ya@ku.th (S.Y.)
[2] Concord College, Acton Burnell Hall, Acton Burnell, Shrewsbury, Shropshire SY5 7PF, UK; 2335017@concordcollege.org.uk
[3] National Nanotechnology Center, National Science and Technology Development Agency, Thailand Science Park, Pathum Thani 12120, Thailand; kantapat.cha@nanotec.or.th
[4] Synchrotron Light Research Institute, 111 University Avenue, Suranaree, Muang, Nakhon Ratchasima 30000, Thailand; suttipong@slri.or.th
[5] Department of Chemistry, Faculty of Science, Kasetsart University, Ladyao, Chatuchak, Bangkok 10900, Thailand; fscinwc@ku.ac.th
[6] School of Materials Science and Innovation, Faculty of Science, Mahidol University, Nakhon Pathom 73170, Thailand; ladda.sth@gmail.com (L.S.); pongsakorn.kan@mahidol.edu (P.K.)
[7] Division of Materials Science, Graduate School of Science and Technology, Nara Institute of Science and Technology, 8916-5 Takayama-cho, Ikoma, Nara 630-0192, Japan; nalin@ms.naist.jp
* Correspondence: fengwwwa@ku.ac.th

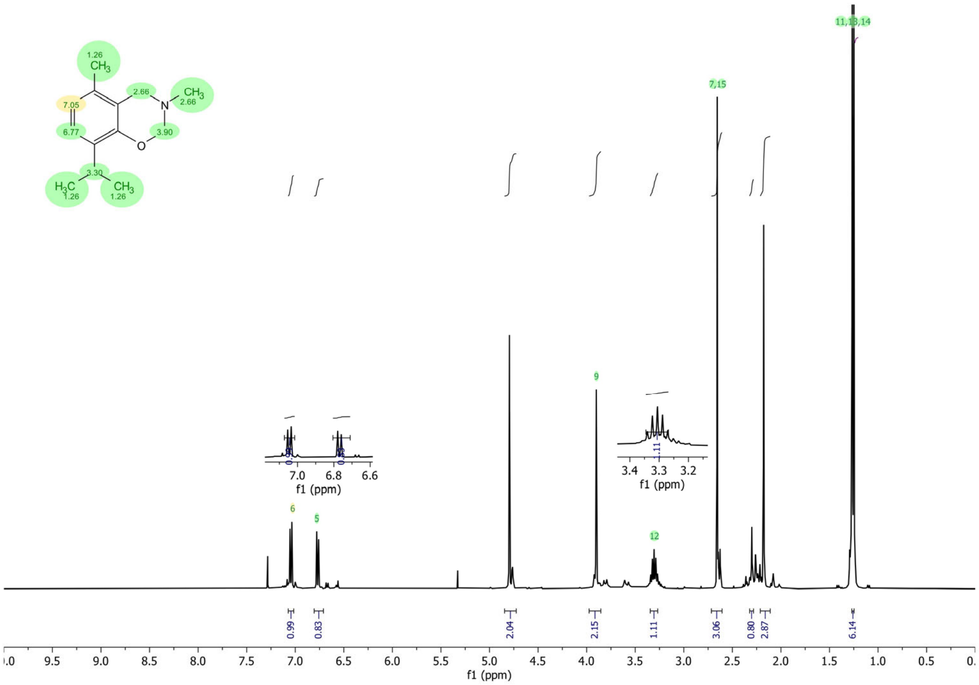

Figure S1. ^1H NMR spectrum of T-m.

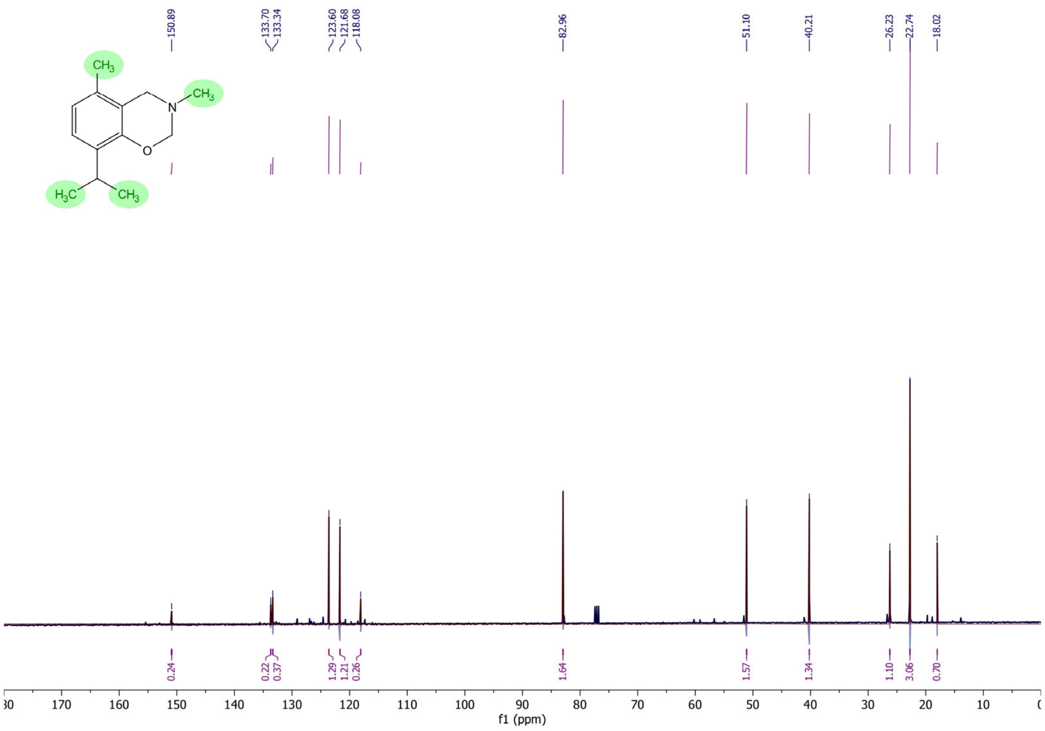

Figure S2. ^{13}C NMR spectrum of T-m.

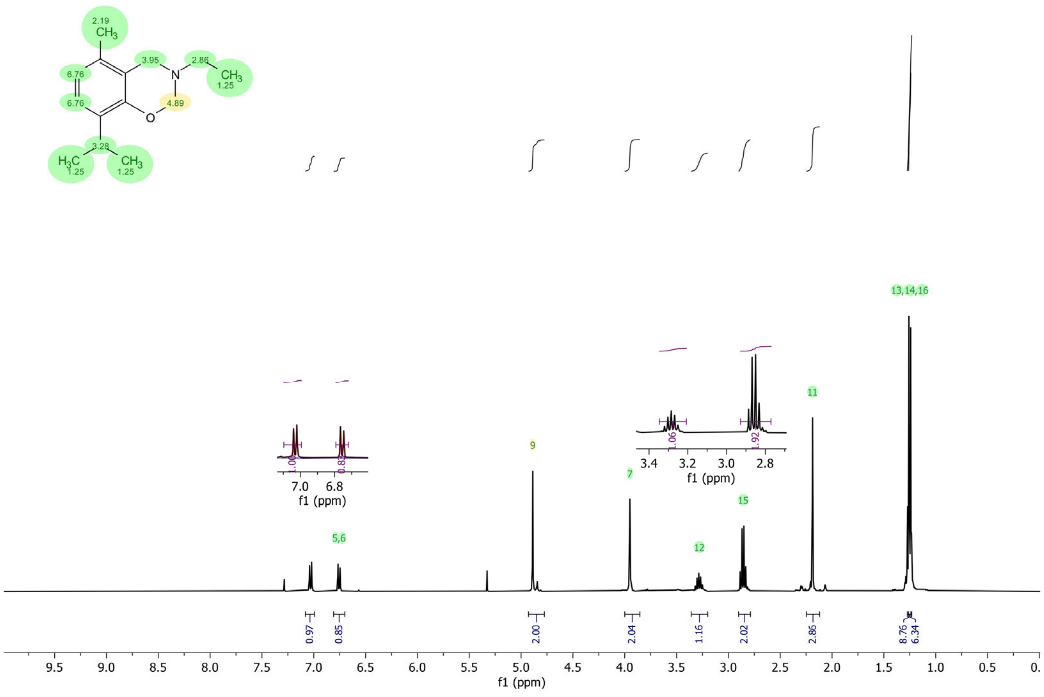

Figure S3. ^{1}H NMR spectrum of T-e.

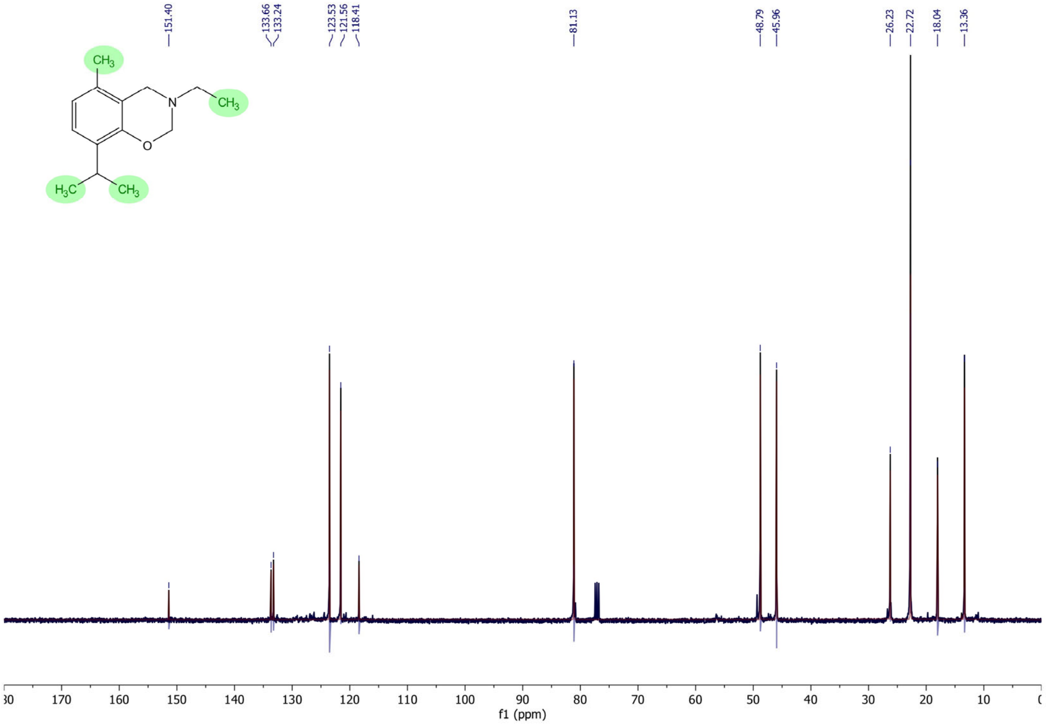

Figure S4. ^{13}C NMR spectrum of T-e.#

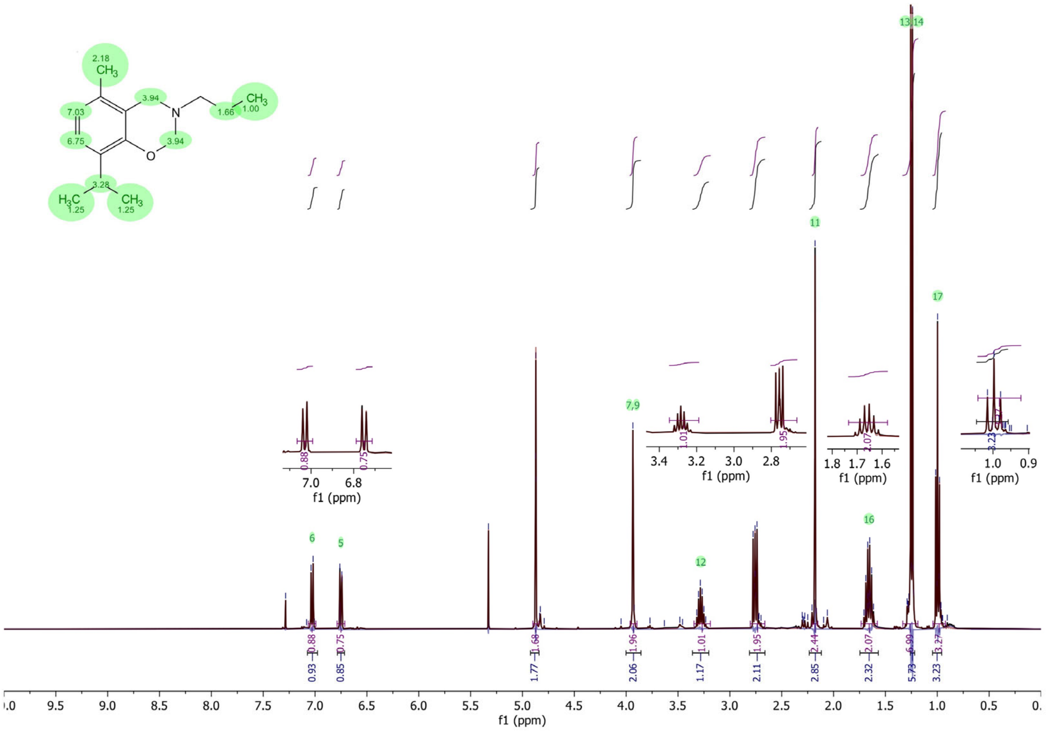

Figure S5. ^{1}H NMR spectrum of T-p.

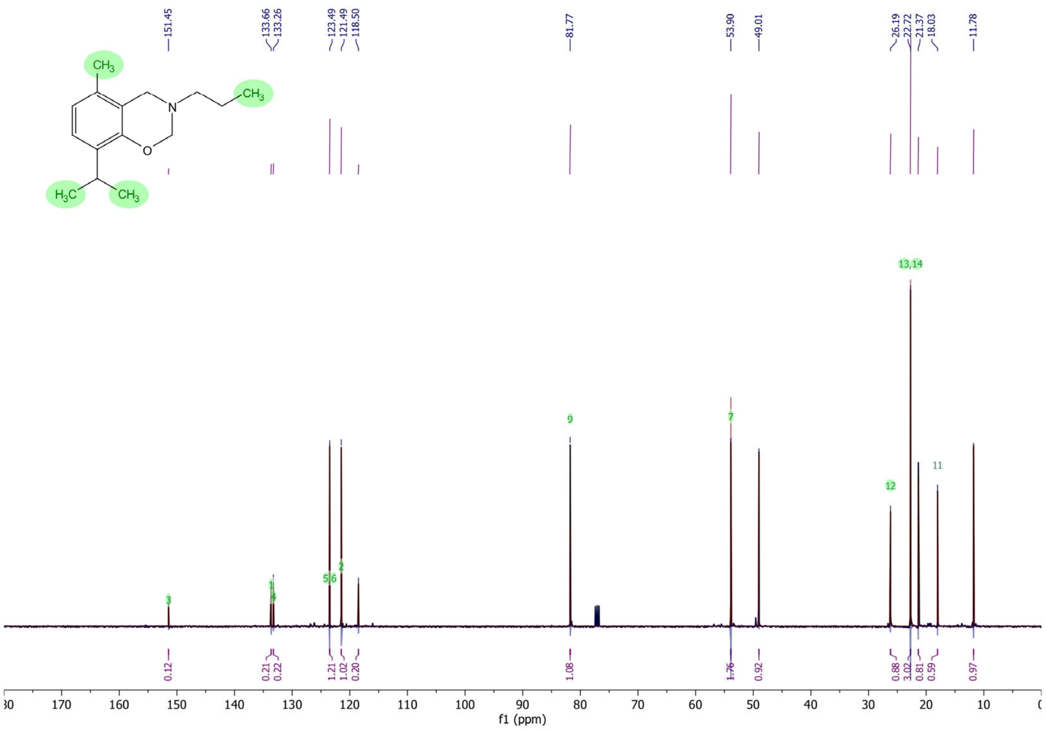

Figure S6. ^{13}C NMR spectrum of T-p.

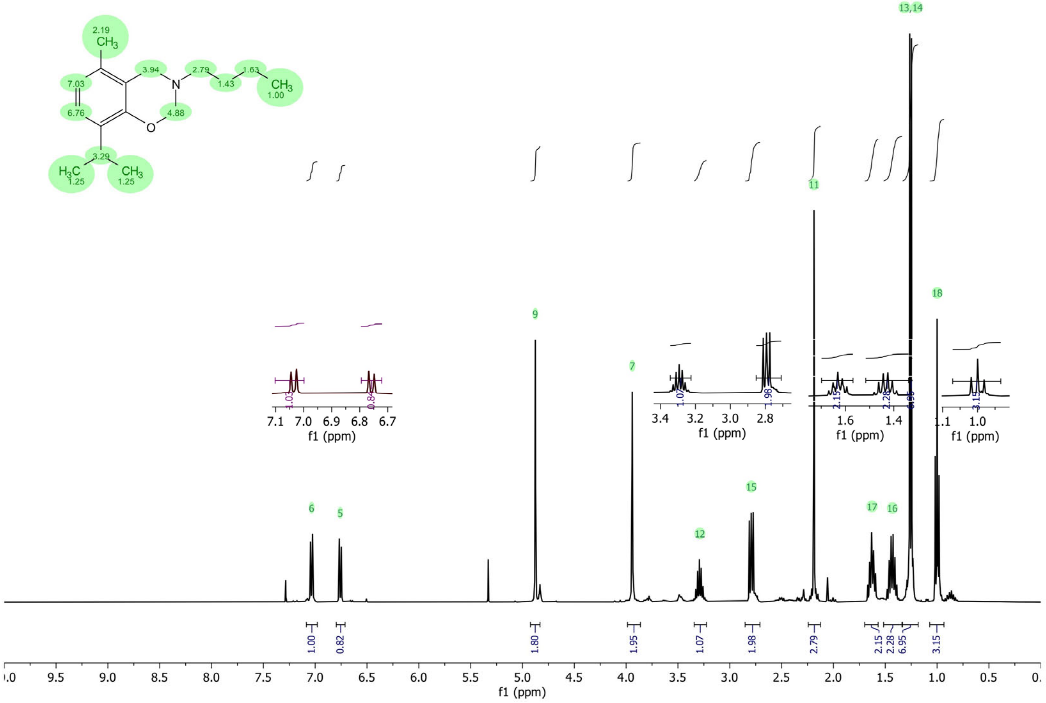

Figure S7. ^{1}H NMR spectrum of T-b.

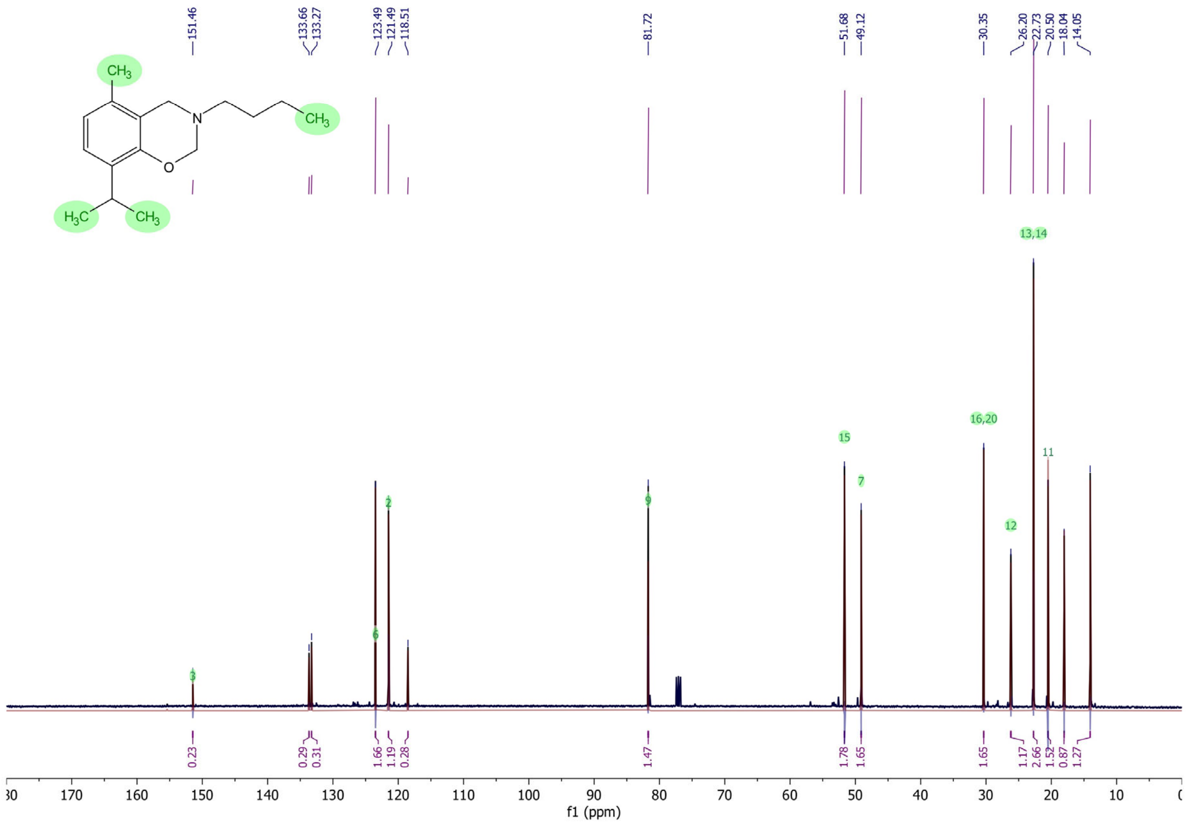

Figure S8. ^{13}C NMR spectrum of T-b.

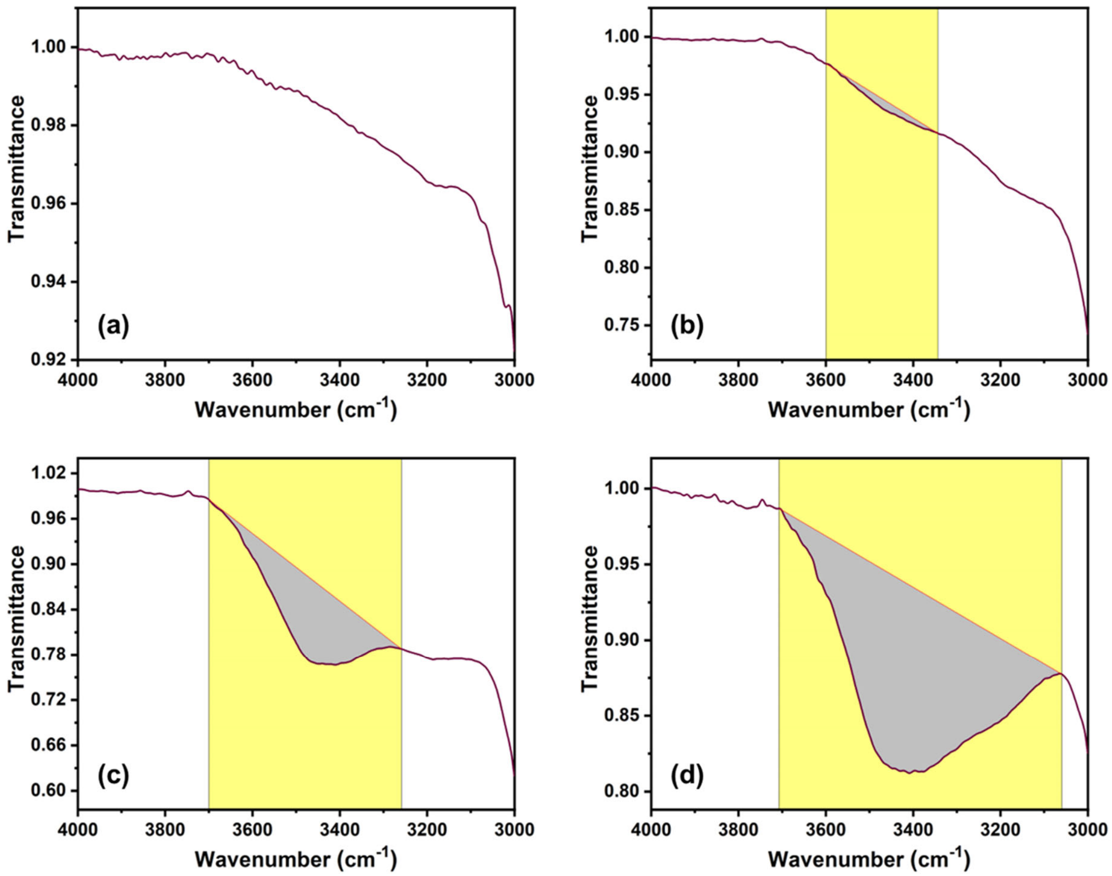

Figure S9. The O–H peak area of (a) T-m, (b) T-m cured at 160 °C, (c) T-m cured at 180 °C, and (d) T-m cured at 200 °C.

Table S1. The O–H peak area of (a) T-m, (b) T-m cured at 160 °C, (c) T-m cured at 180 °C, and (d) T-m cured at 200 °C.

Benzoxazine	Peak Area (a.u.)
T-m	N/A
T-m cured at 160 °C	1.34
T-m cured at 180 °C	21.30
T-m cured at 200 °C	42.61

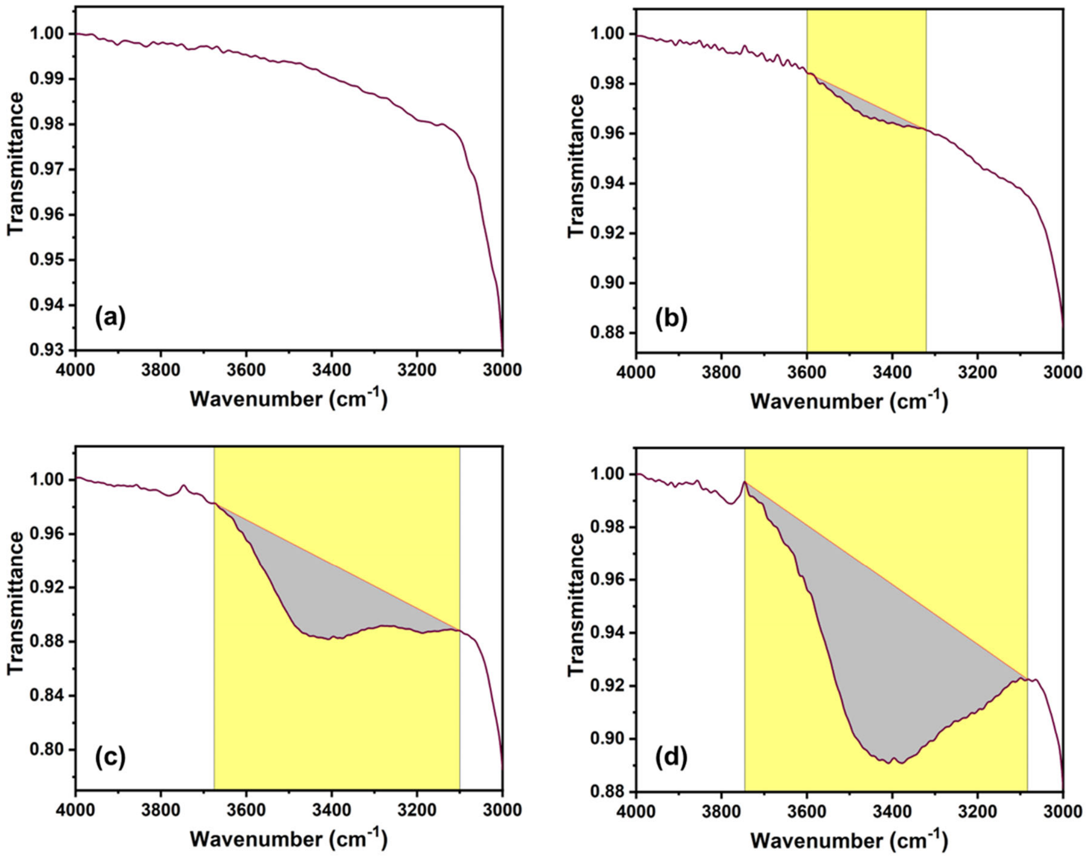

Figure S10. The O–H peak area of (a) T-e, (b) T-e cured at 160 °C, (c) T-e cured at 180 °C, and (d) T-e cured at 200 °C.

Table S2. The O–H peak area of (a) T-e, (b) T-e cured at 160 °C, (c) T-e cured at 180 °C, and (d) T-e cured at 200 °C.

Benzoxazine	Peak Area (a.u.)
T-e	N/A
T-e cured at 160 °C	0.90
T-e cured at 180 °C	17.24
T-e cured at 200 °C	23.61

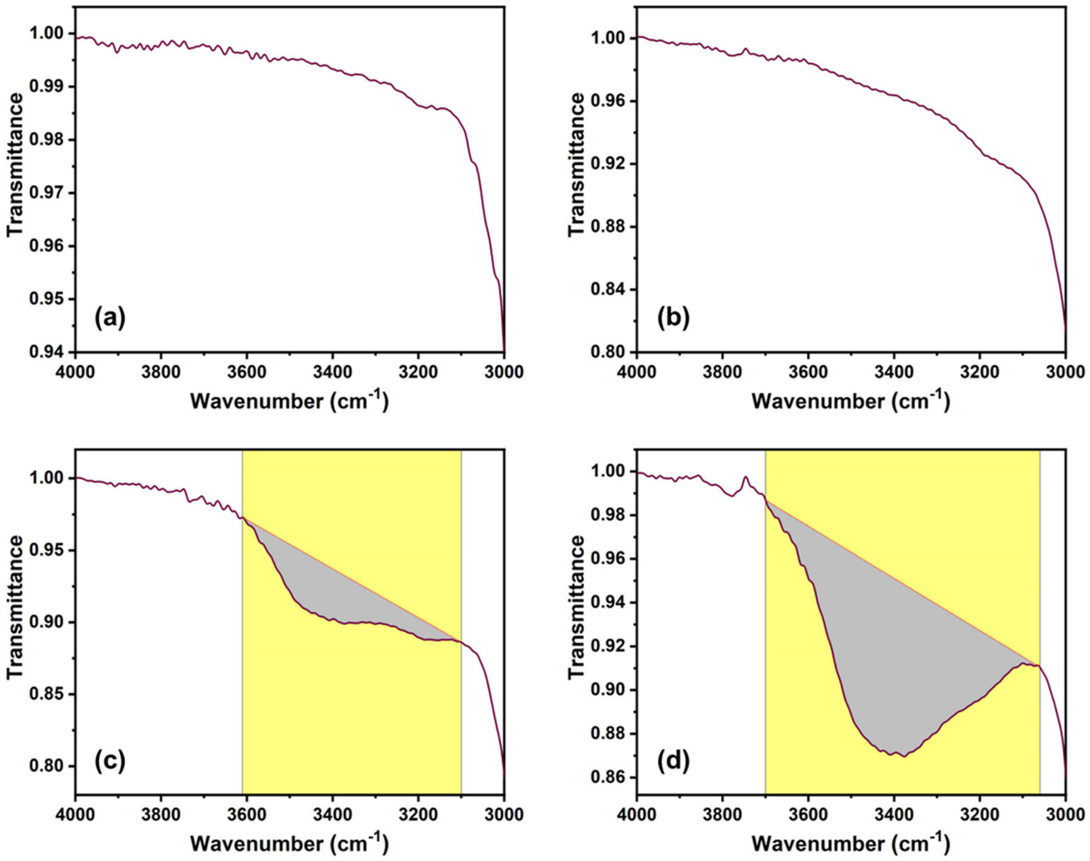

Figure S11. The O–H peak area of (a) T-p, (b) T-p cured at 160 °C, (c) T-p cured at 180 °C, and (d) T-p cured at 200 °C.

Table S3. The O–H peak area of (a) T-p, (b) T-p cured at 160 °C, (c) T-p cured at 180 °C, and (d) T-p cured at 200 °C.

Benzoxazine	Peak Area (a.u.)
T-p	N/A
T-p cured at 160 °C	N/A
T-p cured at 180 °C	10.50
T-p cured at 200 °C	27.16

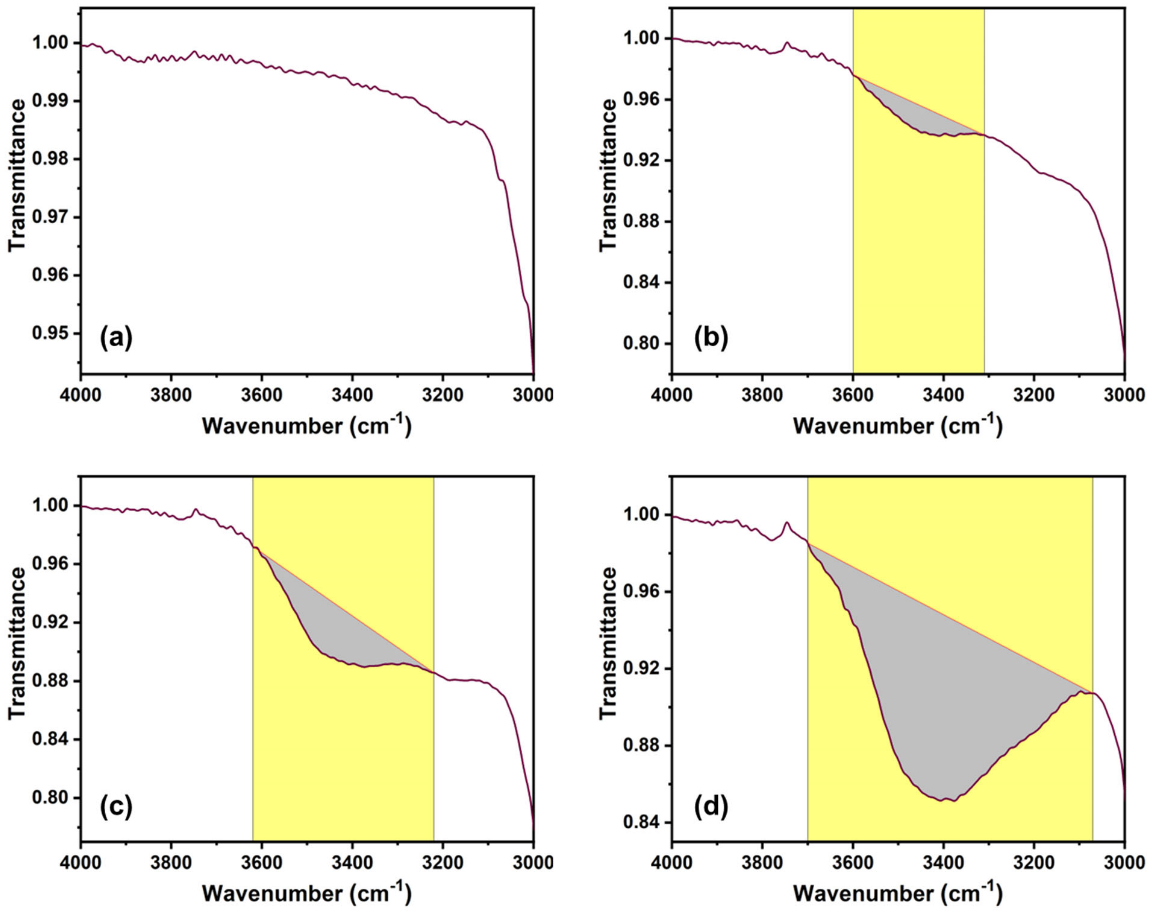

Figure S12. The O–H peak area of (a) T-b, (b) T-b cured at 160 °C, (c) T-b cured at 180 °C, and (d) T-b cured at 200 °C.

Table S4. The O–H peak area of (a) T-b, (b) T-b cured at 160 °C, (c) T-b cured at 180 °C, and (d) T-b cured at 200 °C.

Benzoxazine	Peak Area (a.u.)
T-b	N/A
T-b cured at 160 °C	2.81
T-b cured at 180 °C	8.04
T-b cured at 200 °C	32.27

#
